# Crystal structure of vancomycin bound to the resistance determinant d-alanine-d-serine

**DOI:** 10.1107/S2052252524000289

**Published:** 2024-01-26

**Authors:** Jee Hoon Park, Rachel E. Reviello, Patrick J. Loll

**Affiliations:** a Department of Biochemistry and Molecular Biology, Drexel University College of Medicine, PA 19102, USA; University of Auckland, New Zealand

**Keywords:** vancomycin, vancomycin-resistant enterococci, antibiotics, antibiotic resistance, supramolecular assembly, macromolecular machines, drug discovery, molecular recognition

## Abstract

Vancomycin is a glycopeptide antibiotic that for decades has been a mainstay of treatment for persistent bacterial infections. However, the spread of antibiotic resistance threatens its continued utility. In particular, vancomycin-resistant enterococci (VRE) have become a pressing clinical challenge. Vancomycin acts by binding and sequestering the intermediate Lipid II in cell-wall biosynthesis, specifically recognizing a d-alanine-d-alanine dipeptide motif within the Lipid II molecule. VRE achieve resistance by remodeling this motif to either d-alanine-d-lactate or d-alanine-d-serine; the former substitution essentially abolishes recognition by vancomycin of Lipid II, whereas the latter reduces the affinity of the antibiotic by roughly one order of magnitude. The complex of vancomycin bound to d-alanine-d-serine has been crystallized, and its 1.20 Å X-ray crystal structure is presented here. This structure reveals that the d-alanine-d-serine ligand is bound in essentially the same position and same pose as the native d-alanine-d-alanine ligand. The serine-containing ligand appears to be slightly too large to be comfortably accommodated in this way, suggesting one possible contribution to the reduced binding affinity. In addition, two flexible hydroxyl groups – one from the serine side chain of the ligand, and the other from a glucose sugar on the antibiotic – are locked into single conformations in the complex, which is likely to contribute an unfavorable entropic component to the recognition of the serine-containing ligand.

## Abstract 2

A structure is presented for vancomycin bound to the resistance-associated epitope d-Ala-d-Ser. This high-resolution view of the complex suggests that the reduced affinity of vancomycin for this epitope stems from a subtle size mismatch between the ligand and the antibiotic, in addition to potential unfavorable entropic effects.

## 1. Introduction

The glycopeptide antibiotic vancomycin has been in clinical use since 1958, during which time it has become a valuable therapeutic tool. It is commonly used to treat infections that have responded poorly to other antibiotics, and is also used in patients who cannot tolerate β-lactams (Levine, 2006 ▸). Vancomycin usage increased markedly in the late twentieth century, in response to the appearance of methicillin-resistant *Staphylococcus aureus* (MRSA) and penicillin-resistant *Streptococcus pneumoniae*. Inevitably, increased levels of vancomycin resistance followed, leading to the emergence of vancomycin-resistant pathogens as serious threats to global public health (WHO, 2017 ▸).

**Figure 1:**
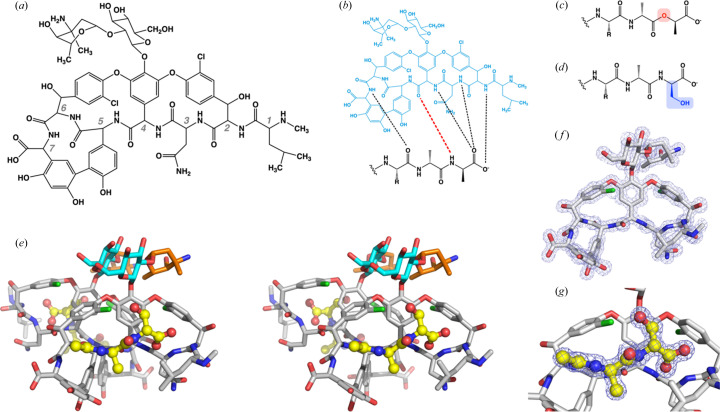
(*a*) Chemical structure of vancomycin. The residue numbering for the amino acids of the glycopeptide is given in gray. (*b*) Schematic representing the interaction of the d-Ala-d-Ala peptide (black) with the antibiotic (blue). Five key hydrogen bonds that link the peptide to the antibiotic are shown as dashed lines. The red dashed line represents the hydrogen bond that is disrupted during binding of the d-Ala-d-Lac ligand. (*c*) Structure of the d-Ala-d-Lac ligand; the oxygen atom of the d-lactate is highlighted in red. (*d*) Structure of the d-Ala-d-Ser ligand; the serine side chain is highlighted in blue. (*e*) Divergent stereoview of a back-to-back vancomycin dimer bound to d-Ala-d-Ser. The dipeptide ligands are shown in ball-and-stick representation. Color scheme: nitro­gen atoms, blue; oxygen atoms, red; chlorine atoms, green. Carbon atoms are yellow for the ligand, gray for the vancomycin aglycon, cyan for the glucose sugar and orange for the vancosamine sugar. (*f*) Representative 2*F*_o_ − *F*_c_ electron density shown for one vancomycin molecule, contoured at 1.6σ. (*g*) 2*F*_o_ − *F*_c_ electron density for one d-Ala-d-Ser ligand, contoured at 1.6σ.

Vancomycin targets biosynthesis of the bacterial cell wall. The antibiotic binds to the so-called muramyl peptide, a component of the biosynthetic intermediate Lipid II; by binding and sequestering this intermediate, vancomycin blocks the late stages in cell-wall production (Williams & Bardsley, 1999 ▸). The precise binding epitope for vancomycin is a conserved d-alanine-d-alanine sequence (d-Ala-d-Ala) that is found at the C-terminus of the muramyl peptide. This epitope is altered in vancomycin-resistant organisms, which have acquired clusters of genes that allow them to alter the makeup of their cell walls (Li *et al.*, 2022 ▸; Stogios & Savchenko, 2020 ▸). These organisms produce variants of Lipid II in which the d-alanine-d-alanine sequence is replaced by either d-alanine-d-lactate (d-Ala-d-Lac) or d-alanine-d-serine (d-Ala-d-Ser). The d-Ala-d-Lac substitution drastically affects the recognition of the muramyl peptide by vancomycin, reducing the affinity of the antibiotic 1000-fold relative to its affinity for the d-Ala-d-Ala-containing species (Walsh *et al.*, 1996 ▸; Bugg *et al.*, 1991 ▸). The ability to incorporate d-Ala-d-Lac into Lipid II is therefore associated with high levels of vancomycin resistance. This resistance mechanism is used by what are currently the most common types of vancomycin-resistant enterococci, types A, B, D and M (Guffey & Loll, 2021 ▸). The pronounced effect of the d-Ala-d-Lac substitution can be rationalized in structural terms, as the amide nitro­gen of the terminal d-Ala residue participates in a hydrogen bond with a carbonyl oxygen on the antibiotic. Changing this NH group (d-Ala) to an oxygen (d-Lac) precludes formation of this hydrogen bond, and additionally places two oxygen atoms in close apposition (Fig. 1 ▸).

In contrast, the d-Ala-d-Ser substitution has a much more modest effect on vancomycin binding, reducing the affinity of the antibiotic for its target by only sevenfold (Billot-Klein *et al.*, 1994 ▸). The d-Ala-d-Ser modification is therefore associated with lower levels of vancomycin resistance; it is currently found in types C, E, G, L and N of vancomycin-resistant enterococci (Reynolds & Courvalin, 2005 ▸). The enteric pathogen *Clostridioides difficile* also carries a vancomycin-resistance gene cluster capable of producing the same modification (Belitsky, 2022 ▸). Given that the d-Ala-d-Ser substitution is associated with relatively small changes in vancomycin susceptibility, organisms expressing this phenotype are typically considered less of a public-health threat than those expressing the d-Ala-d-Lac modification. However, even small reductions in antibiotic susceptibility can confer a survival advantage in the presence of sub-optimal drug doses, providing the opportunity for mutations to accumulate that might ultimately confer higher-level resistance (Shen *et al.*, 2020 ▸). Hence, the d-Ala-d-Ser resistance mechanism cannot be ignored.

In order to provide structural insights into how vancomycin interacts with a serine-containing ligand, we determined a high-resolution X-ray crystal structure of the antibiotic bound to the d-Ala-d-Ser dipeptide. The overall structure of the complex is fundamentally similar to that of vancomycin bound to d-Ala-d-Ala. Hence, the loss of affinity associated with the d-Ala-d-Ser substitution most likely results from subtle structural effects, which include a slight size mismatch between the antibiotic and the ligand, and higher entropic costs associated with immobilizing the d-Ala-d-Ser ligand.

## 2. Materials and methods

To explore d-Ala-d-Ser recognition by vancomycin, we used a synthetic *N*-acetyl­ated dipeptide as a mimic of the muramyl peptide (Biomatik Corporation, Kitchener, Ontario). Dissolving the lyophilized dipeptide in water yielded a strongly acidic solution, presumably reflecting the residual acid remaining in the sample after purification. Mixing this acidic peptide sample with vancomycin gave a clear solution; however, when this solution was tested with a panel of commercially available crystallization screens, no crystals were obtained. We next attempted to bring the pH of the vancomycin–peptide solution closer to neutrality, but this caused rapid precipitation. Adding DMSO as a cosolvent improved the solubility of the complex, but did not eliminate the problem entirely. We therefore tested a wide range of factors for their effects on solubility; these included pH, choice of buffering agent and cosolvent, and the order of addition of the various solution components. Ultimately, we found that DMSO worked well as a cosolvent, and that MES, HEPES and Tris buffers covering the pH range 6–8 could be tolerated. Importantly, we also found that it was necessary to add the various solution components in a specific order. We began with a concentrated vancomycin stock in water (60 mg ml^−1^, *ca* 41 m*M*); we then added DMSO, followed by concentrated buffer, then water and finally the peptide stock. This procedure yielded a clear solution that proved sufficiently stable toward precipitation to allow crystallization experiments to proceed.

For the crystals used to determine the structure, a solution was prepared containing 13.5 m*M* vancomycin and 27 m*M N*-acetyl-d-Ala-d-Ser peptide in 50 m*M* Tris, pH 7.5 and 20%(*v*/*v*) DMSO. 1 µl of this solution was mixed with 1 µl of 25%(*w*/*v*) PEG 1500 in MIB buffer, pH 6 (25 m*M* malonic acid, 37 m*M* imidazole, 37.5 m*M* boric acid), and incubated under Al’s Oil at 4°C (D’Arcy *et al.*, 1996 ▸). Elongated diamond-shaped plates formed in ∼2 weeks. One such crystal was flash-cooled directly in liquid nitro­gen and used for data collection at beamline 17-ID-1 of the National Synchrotron Light Source II (Upton, NY). Raw diffraction data have been deposited with the Zenodo repository, and can be found at https://doi.org/10.5281/zenodo.7650364.

The *Phenix* software suite was used for structure determination and refinement (Liebschner *et al.*, 2019 ▸). A vancomycin structural model was derived from the Cambridge Crystallographic Data Center (model No. 704975) and used for molecular replacement with *Phaser* (McCoy *et al.*, 2007 ▸). Ultimately, five back-to-back vancomycin dimers were placed in the crystal asymmetric unit. The initial model was iteratively adjusted in the graphics program *Coot* (Emsley *et al.*, 2010 ▸) and refined in *Phenix* while adding peptide ligands and solvent molecules. Stereochemical restraints were generated using the *eLBOW* functionality of *Phenix* (Moriarty *et al.*, 2009 ▸), and adjusted where appropriate based on the geometries of previously published atomic resolution structures of vancomycin (Loll *et al.*, 1997 ▸, 2009 ▸; Schäfer *et al.*, 1996*b* ▸; Zarkan *et al.*, 2017 ▸). Data collection and refinement details can be found in Table 1 ▸. Superposition of various structures was carried out using the program *LSQKAB* (Kabsch, 1976 ▸).

## 3. Results and discussion

### 3.1. Overall structure

Molecular replacement was used to determine the structure of the complex of vancomycin with *N*-acetyl-d-Ala-d-Ser, which was then refined at a resolution of 1.20 Å (Fig. 1 ▸; Table 1 ▸). The asymmetric unit contains ten vancomycin molecules, each bound to a d-Ala-d-Ser dipeptide. Each vancomycin monomer adopts a curved structure; the concave faces bind the dipeptide ligands, while the convex faces form dimerization interfaces (Loll & Axelsen, 2000 ▸). These interfaces mediate assembly of the ten vancomycin molecules in the asymmetric unit into five ‘back-to-back’ dimers. Such dimers are ubiquitous in vancomycin crystal structures (Loll *et al.*, 1997 ▸, 2009 ▸, 1999 ▸; Nitanai *et al.*, 2009 ▸; Schäfer *et al.*, 1996*a* ▸; Zarkan *et al.*, 2017 ▸), and also form in solution (Gerhard *et al.*, 1993 ▸). Dimerization is relatively weak in the absence of ligand, but increases cooperatively with ligand binding (Jusuf *et al.*, 2003 ▸; Williams *et al.*, 1998 ▸). In the back-to-back dimer, the ‘front’ of a monomer indicates its concave ligand-binding face, while its ‘back’ is its convex face. Herein, two chains forming a back-to-back dimer will be indicated with a colon; for example, A:B indicates a dimer of chains A and B.

Each vancomycin monomer consists of a macrocyclic peptide core (the aglycon) plus a disaccharide group. The aglycon conformations are very similar for all ten monomers; pairwise superpositioning of all possible monomer combinations yields RMSD values ranging from 0.07 to 0.66 Å for all non-hydrogen atoms of the aglycon (Table S1 of the supporting information). Within each dimer, the aglycons adhere to approximate *C*2 symmetry, whereas the carbohydrate moieties do not, and instead pack against each other in an antiparallel manner. As a consequence, the two ligand-binding sites within each dimer are non-equivalent, with each site having a different sugar overhanging it (Figs. S1 and S2 of the supporting information). Four of the five dimers in the asymmetric unit show a clear preference for the orientation of the carbohydrate moieties, while the fifth has the carbohydrates present in both possible orientations, at approximately equal levels.

**Figure 2:**
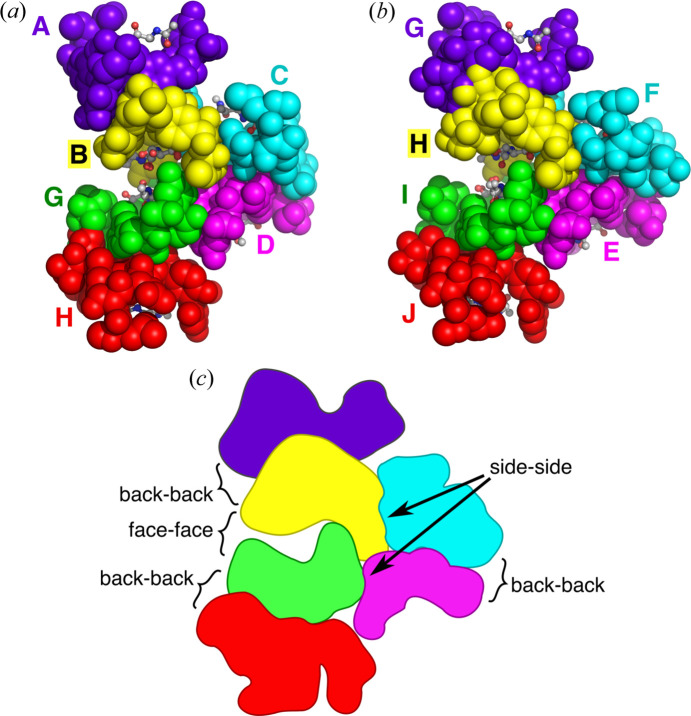
Supramolecular complexes found in the vancomycin–d-Ala-d-Ser crystal structure. (*a*) Supramolecular complex formed from six vancomycin chains and associated ligands: chains A:B, C:D and G:H (two chain names connected by a colon represent a back-to-back complex). (*b*) Supramolecular complex formed from chains E:F, G:H and I:J. (*c*) Schematic showing the different interactions that contribute to the formation of the supramolecular complex. Refer to figures 1 and 2 in Loll *et al.* (2009 ▸) to compare these supramolecular complexes with that formed by the vancomycin–d-Ala-d-Ala complex.

The five dimers in the asymmetric unit are further assembled into two supramolecular structures, each of which contains three vancomycin dimers (one dimer participates in two such structures). Within each of these supramolecular assemblies, two dimers associate face-to-face in a ligand-mediated manner, while a third packs against the side of the face-to-face interface (Fig. 2 ▸). One of the assemblies contains chains A:B, C:D and G:H, while the other contains chains E:F, G:H and I:J. Such supramolecular assemblies have been observed previously, both in crystals and in solution (Lehmann *et al.*, 2002 ▸; Loll *et al.*, 2009 ▸; Nitanai *et al.*, 2009 ▸); they are also observed to form spontaneously in molecular dynamics simulations (Jia *et al.*, 2013 ▸). The assemblies observed in this current crystal structure are highly similar to those reported previously; for example, the two hexameric complexes found in the current structure can be readily superimposed on the equivalent assembly formed by the vancomycin:*N*-acetyl-d-Ala-d-Ala complex, with RMS differences in the Cα positions 0.61 and 0.66 Å. The occurrence of this supramolecular complex in the new d-Ala-d-Ser crystal form underscores its conserved nature.

### 3.2. Ligand recognition and ligand environment

**Figure 3:**
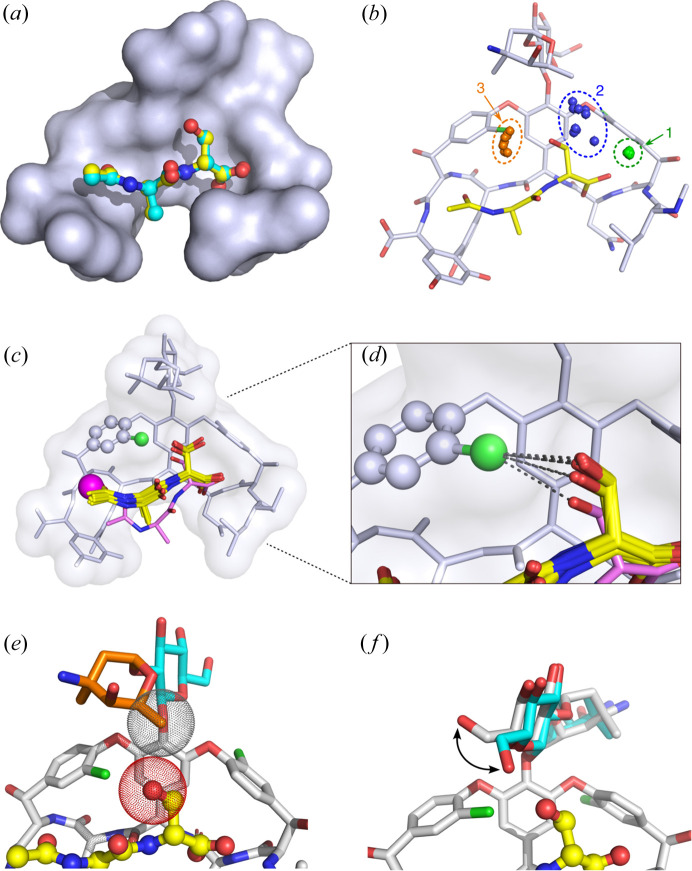
Recognition of the d-Ala-d-Ser ligand. (*a*) Vancomycin’s recognition of d-Ala-d-Ser is virtually the same as its binding of d-Ala-d-Ala. Both ligands are shown superimposed here in ball-and-stick representations; carbon atoms in d-Ala-d-Ser are cyan and in d-Ala-d-Ala are yellow. The vancomycin molecule is shown as a blue–gray surface representation. (*b*) Clusters of conserved water molecules can be observed after superimposing all ten copies of the antibiotic–ligand complex. Waters in cluster 1 (green) form a hydrogen bond with the C-terminal carboxyl­ate of the ligand; those in cluster 2 (blue) hydrogen bond to the serine hydroxyl of the ligand, and those in cluster 3 (orange) form hydrogen bonds to both the serine hydroxyl and the carbonyl oxygen of the penultimate d-Ala residue. (*c*) Nine of the ten copies of the d-Ala-d-Ser ligand adopt essentially identical binding modes. The ten copies of the antibiotic–ligand complex were superposed using *LSQKAB* (Kabsch, 1976 ▸); only one copy of the antibiotic is shown for clarity. The nine copies of the ligand that adopt similar poses are shown as yellow sticks; the outlier is shown in magenta. The magenta sphere represents a water molecule found in the binding site next to the outlier ligand; this water occupies the position that is filled by the acetyl oxygen in the other nine copies of the antibiotic–ligand complex, and forms a hydrogen bond with an amide nitro­gen on the antibiotic. (*d*) Enlarged view of the d-Ser ligands shows halogen bonds (dashed lines) formed between the chlorine atom of residue 6 of the antibiotic (green sphere) and the oxygen atom of the serine hydroxyl group. (*e*) Close contact between the serine hydroxyl of the ligand and the C5 methyl group of the antibiotic vancosamine sugar. The van der Waals radii for the oxygen and carbon atoms are shown as dot surfaces. (*f*) The presence of the d-Ser side chain (yellow) forces a rotation of the C6 hydroxyl group of the antibiotic’s glucose sugar. The glucose conformation shown in gray represents what is observed in the presence of the serine-containing ligand; cyan shows an alternative conformation that is observed with smaller ligands, but which cannot form in the presence of d-Ser.

For all ten copies of the vancomycin:d-Ala-d-Ser complex, the antibiotic’s mode of ligand recognition is similar to its recognition of the native d-Ala-d-Ala ligand (Loll *et al.*, 2009 ▸). For nine of the ten antibiotic–ligand complexes, the d-Ala-d-Ser dipeptide occupies the same position and adopts essentially the same pose as d-Ala-d-Ala, and forms the same five hydrogen bonds with the antibiotic [Figs. 3 ▸(*a*) and 3 ▸(*c*)]. The C-terminal d-Ser of the tenth ligand is situated similarly to the serine residues of the other nine ligands, allowing it to form four of the five canonical hydrogen bonds with the antibiotic; however, the N-terminus of the tenth ligand is displaced outward, away from the antibiotic, preventing formation of the fifth hydrogen bond, which would normally link the carbonyl oxygen of the *N*-acetyl group to the antibiotic. Instead, this bond is replaced by a hydrogen bond between the antibiotic and a water molecule [Fig. 3 ▸(*c*)]. Overall, despite this one instance of conformational heterogeneity, the presence of the serine side chain does not appear to disrupt the essential architecture of ligand recognition.

Changing the ligand from d-Ala-d-Ala to d-Ala-d-Ser introduces a new rotatable bond, which prompted us to examine the rotameric state of the serine side chain. Of the three possible serine rotamers, the *gauche* rotamer with χ_1_ = 60° is the most preferred, accounting for roughly 45% of serine residues found in protein structures (Dunbrack & Karplus, 1993 ▸). The other *gauche* rotamer (χ_1_ = −60°) and the *trans* rotamer (χ_1_ = 180°) are less common, accounting for 30 and 25% of serine residues, respectively. In the structure reported here, χ_1_ values fall into the range 68–75° for all ten copies of the ligand, corresponding to the *gauche* (+60°) rotamer; importantly, the *gauche* (−60°) rotamer cannot be accommodated in the complex, as it would lead to a steric clash between the serine hydroxyl and residue 4 of the vancomycin molecule. Eight of the ten ligands in the asymmetric unit adopt only the *gauche* (+60°) rotamer; the remaining two copies also adopt an alternate *trans* conformation, with the *guache* and *trans* rotamers being present with roughly equal occupancies. The two ligands that adopt alternate conformers are both found in face-to-face antibiotic interfaces; this positioning allows a resorcinol hydroxyl group from a neighboring vancomycin molecule to form a hydrogen bond with the *trans* rotamer of the serine, which presumably explains why this rotamer is seen only in this location.

In addition to forming hydrogen bonds with adjoining antibiotic molecules, the serine side chain of the ligand also interacts with water molecules in the local environment. Nine of the ten ligand molecules each form hydrogen bonds with two waters via their serine side chains, while the tenth forms a single hydrogen bond with water. The positions of these waters relative to the serine hydroxyl are well conserved, suggesting that they represent firmly held inner-sphere solvent molecules [Fig. 3 ▸(*b*)].

The serine side chain of the ligand also forms a halogen bond with the chlorine atom found on residue 6 of the antibiotic [Fig. 3 ▸(*d*)]. For all of the ten ligands, the oxygen–chlorine distance is less than or equal to 3.27 Å, which is the sum of the van der Waals radii for the two elements. The actual distances vary for the different copies of the antibiotic–ligand complex, ranging from 3.0 to 3.27 Å. The halogen bond (Cl⋯O) is nearly co-linear with the C—Cl bond that connects the chlorine atom to the aromatic ring, with C—Cl⋯O angles ranging from 159 to 178°. This geometry is consistent with the conformations of halogen bonds observed in small-molecule crystal structures (Cody & Murray-Rust, 1984 ▸; Lommerse *et al.*, 1996 ▸; Ouvrard *et al.*, 2003 ▸), and is also in agreement with interaction geometries predicted from electrostatic calculations (Auffinger *et al.*, 2004 ▸).

Finally, binding of the d-Ala-d-Ser ligand affects the conformations of the antibiotic carbohydrate groups. As discussed earlier, each vancomycin dimer contains two non-equivalent ligand-binding sites, with either a vancosamine or a glucose sugar extending over the ligand. In the sites overhung by a vancosamine, the C5 methyl group of the sugar sits directly over the d-Ser side chain of the ligand, forming part of a shallow hydro­phobic pocket into which the β-carbon of the ligand can pack. This places the C5 methyl group close to the hydroxyl oxygen of serine, with carbon–oxygen distances ranging from 3.1 to 3.8 Å [Fig. 3 ▸(*e*)]. For the binding sites overhung by a glucose, the carbohydrate similarly sits overtop of the ligand serine hydroxyl, with the top of the binding pocket being formed by the C5 and C6 atoms of the sugar. These two carbon atoms occupy approximately the same position as that occupied by the C5 methyl group of vancosamine, but they do not approach quite so closely to the hydroxyl oxygen. The C6 hydroxyl group of the glucose rotates upward, away from the binding site, in order to avoid a clash with the serine side chain of the ligand [Fig. 3 ▸(*f*)]. In vancomycin complexes with smaller ligands, no such clash occurs, and this hydroxyl group can adopt a different rotamer that places the oxygen of the hydroxyl within halogen-bonding distance of the chlorine atom on residue 6 (Loll *et al.*, 1997 ▸, 2009 ▸, 1999 ▸).

### 3.3. Discussion

Vancomycin and related glycopeptide antibiotics recognize the muramyl peptide ligand using a conserved binding mode that relies on five ligand–antibiotic hydrogen bonds, as well as a van der Waals contact between the side chain of the ligand’s terminal d-alanine and an aromatic ring on the antibiotic (Fig. 1 ▸; Loll & Axelsen, 2000 ▸). Highly detailed views of the antibiotic–ligand interaction have been obtained from high-resolution crystal structures, but NMR studies indicate that ligand recognition in solution is essentially similar to that observed in crystals (Barna & Williams, 1984 ▸; Molinari *et al.*, 1990 ▸). Small apparent differences in the ligand-binding mode have been noted, but are now thought to reflect artifacts stemming from conformational fluctuations in solution and interpretation of the NMR data (Jia *et al.*, 2013 ▸).

To subvert the vancomycin ligand-recognition mechanism and thus evade the antibiotic toxicity, bacteria incorporate the d-Ala-d-Ser sequence into their muramyl peptides in order to reduce the binding affinity of the drug. We chose to determine the structure of the vancomycin–d-Ala-d-Ser complex in hopes of elucidating this decreased affinity.

At first glance, the antibiotic recognition of the d-Ala-d-Ser ligand is strikingly similar to its recognition of the native ligand d-Ala-d-Ala. The poses of the two dipeptides within the antibiotic ligand-binding site are essentially identical. Hence, the thermodynamic determinants that confer high-affinity binding on d-Ala-d-Ala are expected to be largely conserved in the d-Ala-d-Ser complex. Further, the antibiotic selects the most favored side-chain rotamer of the serine residue, and the hydroxyl group of this side chain engages in a halogen bond with a chlorine atom of the antibiotic. These factors would seem to suggest that the d-Ala-d-Ser peptide should bind at least as strongly as d-Ala-d-Ala; given the weaker binding affinity of d-Ala-d-Ser, other factors must be at play, providing unfavorable contributions that outweigh these favorable effects.

One such factor is likely to be the entropic cost of immobilizing the serine side chain of the ligand. Even though the antibiotic–peptide complex selects the preferred rotamer of the side chain, there will still be an entropic penalty associated with locking the side chain into a single conformation. An additional unfavorable entropic effect can be found in the interaction between the glucose sugar of the antibiotic and the ligand. In those d-Ala-d-Ser complexes in which the glucose sugar overhangs the binding site, the C6 hydroxyl group of the carbohydrate must rotate up and away from the ligand to avoid a steric clash. In contrast, when the antibiotic binds to smaller ligands such as d-Ala-d-Ala, this hydroxyl group enjoys greater conformational freedom. Hence, the d-Ala-d-Ser ligand limits the conformational space available to this sugar, which presumably incurs an additional entropic cost.

Another possible unfavorable factor relates to the interaction between the d-Ala-d-Ser ligand and the antibiotic vancosamine sugar. The serine hydroxyl of the ligand closely approaches the C5 methyl group of the sugar, and in some of the complexes found in the asymmetric unit, this distance is unfavorably close. The shortest carbon–oxygen distance observed is 3.1 Å, which is less than the sum of the van der Waals radii (3.22 Å). This difference (3.1 versus 3.22 Å) is close to the maximum-likelihood estimate for the overall coordinate error in this structure (0.1 Å), so it is unclear whether the two atoms are in fact close enough to give rise to a strong repulsive interaction; however, it is certainly plausible that wedging the serine side chain into this tight pocket exacts at least a small energetic cost.

Finally, we note that while the d-Ala-d-Ser ligand does engage in a halogen bond with a chlorine atom on vancomycin, the halogen-bonding potential of this chlorine atom can also be satisfied internally when smaller ligands are bound. For example, in the complex with d-Ala-d-Ala, the glucose hydroxyl group of the antibiotic can rotate downward towards the ligand-binding pocket to form a halogen bond with this chlorine atom. Thus, the antibiotic–ligand halogen bond observed in the d-Ala-d-Ser complex will not necessarily provide a net gain in binding energy, relative to that of the d-Ala-d-Ala complex.

In summary, the high-resolution structure of vancomycin bound to d-Ala-d-Ser reveals a complex that is highly similar to the antibiotic’s complex with its native ligand d-Ala-d-Ala, with no gross differences in conformation of either the antibiotic or the peptide component. It therefore appears that the reduction in binding affinity observed for the d-Ala-d-Ser ligand results from one or more subtle effects, including the increased entropic cost of binding the serine-containing peptide and a binding site that is slightly too small to comfortably accommodate the hydroxyl group of the ligand.

## Supplementary Material

Supporting table and figures. DOI: 10.1107/S2052252524000289/be5293sup1.pdf

Raw diffraction data and XDS input data for the complex of vancomycin with N-acetyl-D-Ala-D-Ser: https://10.5281/zenodo.7650364

PDB reference: 8g82, vancomycin bound to d-Ala-d-Ser

## Figures and Tables

**Table 1:** Data collection and refinement statistics

Data collection statistics	
PDB entry	8g82
Diffraction source	Beamline 17-ID-1, NSLS-II
Wavelength (Å)	0.9213
Temperature (K)	100
Detector	Dectris EIGER 9M
Resolution range (Å)†	26.5–1.20 (1.24–1.20)
Space group	*P*2_1_2_1_2_1_
Unit cell	
*a*, *b*, *c* (Å)	30.49, 71.15, 82.44
α, β, γ (°)	90.0, 90.0 90.0
No. of observed reflections	276753 (10882)
No. of unique reflections	53037 (3474)
Average multiplicity	5.2 (3.1)
Completeness (%)	93.0 (61.9)
Mean *I*/σ(*I*)	13.5 (1.8)
Estimated Wilson *B* factor (Å^2^)	13.6
*R*_merge_ ‡	0.056 (0.657)
*R*_meas_ §	0.062 (0.777)
*R*_pim_ ¶	0.026 (0.405)
CC_1/2_ ††	0.999 (0.668)

Refinement and model statistics	
No. of reflections used	53028
No. of reflections used for *R*_free_	2000
*R*_work_	0.153 (0.233)
*R*_free_	0.171 (0.292)
No. of non-hydrogen atoms	
Vancomycin	1010
Ligand	150
Solvent	288
Average *B* factor (Å^2^)	
Vancomycin	15.2
Ligand	15.5
Solvent	30.3
RMS deviations from ideality	
Bonds (Å)	0.012
Angles (°)	1.76
Clashscore	2.05

† Values in parentheses refer to the highest-resolution shell.

‡ *R*_merge_ is calculated by the equation *R*_merge_ = Σ*_hkl_* Σ*_i_* |*I_i_* (*hkl*) − 〈*I*(*hkl*)〉|/Σ*_hkl_* Σ*_i_* *I_i_* (*hkl*), where *I_i_* (*hkl*) is the *i*th measurement.

§ *R*_meas_ (or redundancy-independent *R*_merge_) is calculated by the equation *R*_meas_ = Σ*_hkl_* [*N*/(*N* − 1)]^1/2^Σ*_i_* |*I_i_* (*hkl*) − 〈*I*(*hkl*)〉|/Σ*_hkl_* Σ*_i_* *I_i_* (*hkl*), where *I_i_* (*hkl*) is the *i*th measurement and *N* is the redundancy of each unique reflection *hkl* (Diederichs & Karplus, 1997 ▸).

¶ *R*_pim_ is calculated by the equation *R*_pim_ = Σ_*hkl*_[1/(*N* − 1)]^1/2^Σ*_i_* |*I_i_* (*hkl*) − 〈*I*(*hkl*)〉|/Σ*_hkl_* Σ*_i_* *I_i_* (*hkl*), where *I_i_* (*hkl*) is the *i*th measurement and *N* is the redundancy of each unique reflection *hkl* (Weiss, 2001 ▸).

†† CC_1/2_ is the correlation coefficient between two randomly chosen half datasets (Karplus & Diederichs, 2012 ▸).
