# Supplementary material for: Crystal structure of vancomycin bound to the resistance determinant d-alanine-d-serine
Source: IUCrJ. 2024 Jan 26;11(Pt 2):133–9. doi: 10.1107/S2052252524000289 (PMC10916290; doi:10.1107/S2052252524000289)
Supplement: Supplementary file 1 [file m-11-00133-sup1.pdf]

IUCrJ

Volume 11 (2024)

Supporting information for article:

Crystal structure of vancomycin bound to the resistance determinant d-alanine-d-serine

Jee Hoon Park, Rachel E. Reviello and Patrick J. Loll

Table S1 Structural similarity between the ten vancomycin monomers within the asymmetric unit. Pairwise superpositions were carried out for all vancomycin monomers in the asymmetric unit (denoted as chains A-J). Shown are RMSD values (Å) for all non-hydrogen atoms within the vancomycin aglycon after superposition. Carbohydrate moieties were omitted from the superposition and RMSD calculations.

Chain	A	B	C	D	E	F	G	H	I	J
A	---									
B	0.59	---								
C	0.30	0.55	---							
D	0.54	0.62	0.62	---						
E	0.44	0.52	0.49	0.20	---					
F	0.58	0.63	0.63	0.08	0.23	---				
G	0.58	0.09	0.55	0.60	0.50	0.62	---			
H	0.58	0.12	0.56	0.61	0.50	0.62	0.07	---		
I	0.60	0.11	0.57	0.62	0.51	0.63	0.08	0.12	---	
J	0.53	0.61	0.61	0.18	0.26	0.20	0.60	0.60	0.62	---

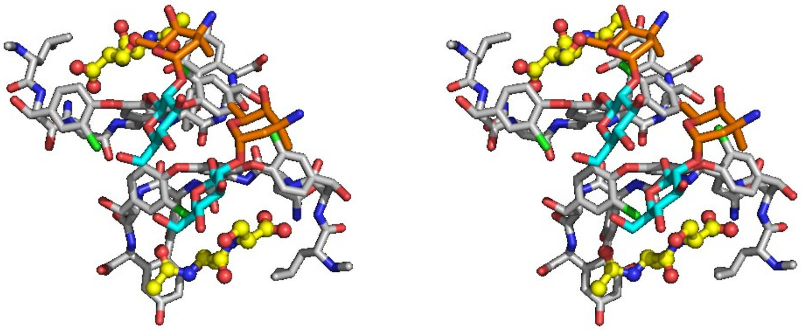

Figure S1 Divergent stereo view of a back-to-back vancomycin dimer bound to D-Ala-D-Ser. This figure uses the same color scheme as Figure 1(*e*); the view is rotated by 90° about a horizontal axis relative to that of Figure 1(*e*). An approximate two-fold axis of symmetry perpendicular to the plane of the page relates the two vancomycin aglycon moieties; the carbohydrate groups do not obey this symmetry.

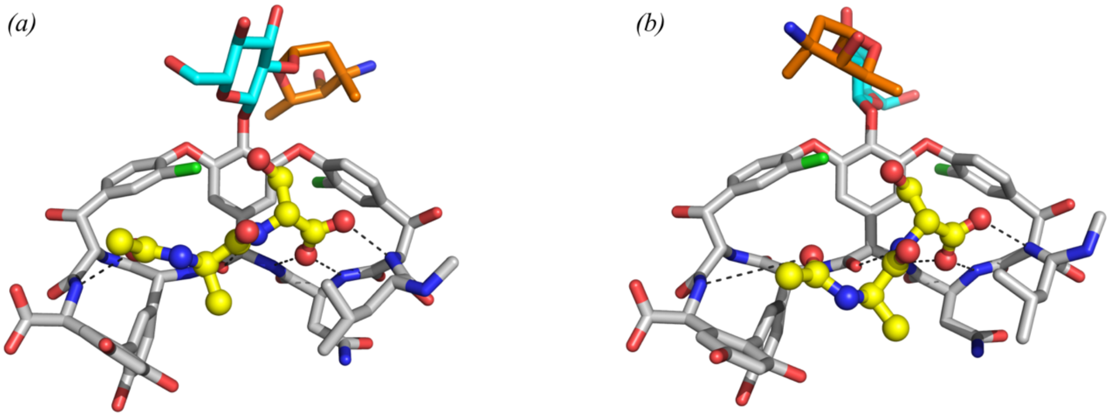

Figure S2 Views looking into the ligand-binding site for the two conformations of the vancomycin carbohydrate group. (*a*) Binding site overhung by glucose; (*b*) binding site overhung by vancosamine. Dashed lines show hydrogen bonds between the ligand and the antibiotic. This figure uses the same color scheme as Figure 1(*e*).
